# Hydrogels as Porogens for Nanoporous Inorganic Materials

**DOI:** 10.3390/gels4040083

**Published:** 2018-10-10

**Authors:** Christian Weinberger, Dirk Kuckling, Michael Tiemann

**Affiliations:** 1 Department of Chemistry—Inorganic Functional Materials, Paderborn University, 33098 Paderborn, Germany; christian.weinberger@uni-paderborn.de; 2 Department of Chemistry—Organic and Macromolecular Chemistry, Paderborn University, 33098 Paderborn, Germany

**Keywords:** nanoporous, mesoporous, metal oxide, silica, hydrogel, thin film, porogen, template, nanocasting

## Abstract

Organic polymer-hydrogels are known to be capable of directing the nucleation and growth of inorganic materials, such as silica, metal oxides, apatite or metal chalcogenides. This approach can be exploited in the synthesis of materials that exhibit defined nanoporosity. When the organic polymer-based hydrogel is incorporated in the inorganic product, a composite is formed from which the organic component may be selectively removed, yielding nanopores in the inorganic product. Such porogenic impact resembles the concept of using soft or hard templates for porous materials. This micro-review provides a survey of select examples from the literature.

## 1. Introduction

Inorganic materials, with uniform nanopores (pore width ≤ 100 nm [1]), play an important role in a large variety of applications, including catalysis [2,3], energy storage [4,5], sensors [6,7], controlled drug release [8,9], biomaterials [10,11], and separation [12,13]. There are various chemical synthesis methods for porous materials that include distinct strategies for the formation of nanopores in the desired size. Among these, sol-gel chemistry-based methods are the most frequent ones. Standard sol-gel protocols yield materials with, more or less, high degrees of porosity [14], but usually with a broad pore size distribution. Extending the sol-gel approach by introducing porogenic ‘soft templates’, such as organic molecules or molecular ions (especially for microporous zeolites [15,16]) or supramolecular arrays of self-assembled amphiphilic species (surfactants or block co-polymers for ordered mesoporous materials [17,18,19]) allows for controlling the pore size and size uniformity (By definition, micropores have widths up to 2 nm; mesopore widths range from 2 to 50 nm [1]). The ‘soft template’ is incorporated in the inorganic solid during its synthesis under sol-gel-chemical conditions, thereby serving as a pore filler and often also as a structure director; it is later removed (by thermal combustion or by ion exchange), yielding uniform nanopores. Utilization of amphiphilic porogens also facilitates the synthesis of mesoporous inorganic materials in form of films by evaporation-induced self-assembly (EISA) [20,21]. However, the ‘soft templating’ approach cannot be regarded as a uniform synthesis method for mesoporous materials; in many cases, the formation of the respective product (such as most metal oxides) goes along with phase-separation and segregation from the amphiphilic species. For that reason, the concept of ‘nanocasting’ or ‘hard templating’ is frequently used for mesoporous materials as an alternative to the ‘soft templating’ method. Here a solid, porous silica or carbon matrix serves as a structural mold. The desired product is synthesized within the pores of the matrix, followed by selective removal of the latter; the product is obtained as a ‘negative replica’ of the pore system in the matrix [18,22,23]. The nanocasting approach may be regarded as a more universal synthesis concept than ‘soft templating’, as the risk of phase separation is avoided.

More recently, organic polymer-based hydrogels have been explored as porogenic matrices for the synthesis of inorganic mesoporous materials. A hydrogel is a network of polymer chains that are hydrophilic and are cross-linked either chemically (by covalent or ionic bonds) or physically (by steric entanglement). Upon exposure to water hydrogels ‘swell’, i.e., take up large quantities of water in the spaces between the polymer strands [24]. The aqueous phase of the swollen gel can be used for the synthesis of an inorganic solid (by sol-gel-like methods or simply by precipitation). The product will then contain the organic polymer matrix embedded in the inorganic material. When the matrix is later removed (e.g., by combustion), it will leave behind a network of mesopores. This porogenic impact of the hydrogel may be regarded as an intermediate between ‘soft’ and ‘hard templating’. One the one hand, the hydrogel is a pre-formed continuous network, similar to a ‘hard’ structure matrix; on the other hand, the swollen hydrogel is very flexible, similar to a ‘soft’ matrix. The general concept of hydrogels serving as matrices for inorganic (though not necessarily porous) phases is also known from biomineralization [25] and tissue engineering [26]. This micro-review gives some examples of the concept of using hydrogels as porogenic matrices for the synthesis of inorganic porous materials.

## 2. Physically Cross-Linked Porogenic Hydrogels

In the literature, there are several reports on the utilization of non-chemically cross-linked organic polymers serving as matrices for inorganic materials. Quite often the polymers are not actually arranged in three-dimensional hydrogel networks, but rather, form fibrous entities that serve as gelators for inorganic nanostructures with a limited degree of porosity. Also, the reaction medium is often an organic solvent rather than water. For example, fibrous SiO_2_ [27,28,29,30] and TiO_2_ [31,32] nanostructures have been synthesized by using a variety organogelators, including chiral compounds for products with helical morphology [28,29]. Sanchez et al. used 2,3-Bis(n-decyloxy)anthracene (DDOA, a typical low-molecular-mass organogelator) for the synthesis of porous alumina (Al_2_O_3_) in a water/ethanol mixture [33]. The products showed a fibrous morphology and (after calcination, i.e., combustion of the organic component) bimodal micro-and mesoporosity. Tang et al. also prepared porous Al_2_O_3_ with high porosity by using an agarose hydrogel [34]. Further examples of low-molecular-mass gelators for inorganic materials with fibrous morphology were reported by Lu et al. who used β-d-Glucopyranoside-functionalized thiosemicarbazides for CdS [35] and *N*-Lauroyl-l-glutamic acid for CuS [36].

**Figure 1:**
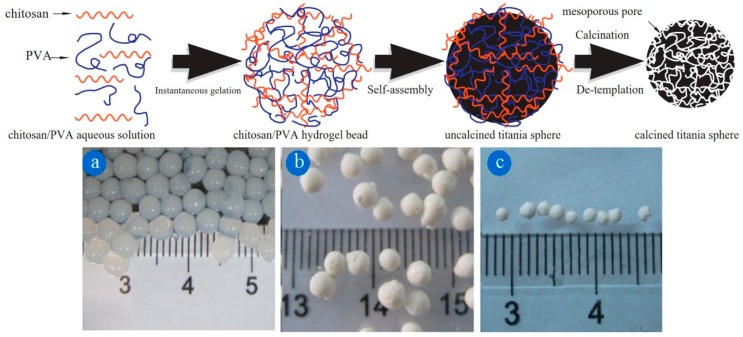
**Top**: schematic of the synthesis of mesoporous TiO_2_ spheres by using a physically cross-linked chitosan/poly(vinyl alcohol) hydrogel as a porogenic matrix. **Bottom**: optical photographs of (**a**) hydrogel beads, (**b**) hydrogel/TiO_2_ composite beads, and (**c**) TiO_2_ beads. Reprinted with permission from [37], Copyright 2014 Elsevier.

A chitosan/poly(vinyl alcohol) hydrogel was used by Zhu and Xu et al. for the synthesis of mesoporous titania (TiO_2_) to be used as photo-catalytically active material [37]. Chitosan and poly(vinyl alcohol) spontaneously form spherical hydrogel beads in water; these served as matrices for millimeter-sized TiO_2_ spheres (Figure 1). After drying, but before removal of the matrix, the TiO_2_ product exhibited mesopores with uniform sizes of ca. 5 nm. Removal of the matrix by calcination led to a gradual decrease in porosity due to structural collapse.

We have recently prepared mesoporous alumina (γ-Al_2_O_3_) and magnesia (MgO) by using dimethylacrylamide-based hydrogels, both with [see below] and without chemical cross-linking [38]. The alumina products had pore sizes in the range between 3.6 to nm and 6.4 nm specific pore volumes up to 0.54 cm^3^ g^−1^; Magnesium oxide exhibited mesopore sizes of 3.6 nm and a lower specific pore volume of ca. 0.37 cm^3^ g^−1^.

## 3. Chemically Cross-linked Porogenic Hydrogels

**Figure 2:**
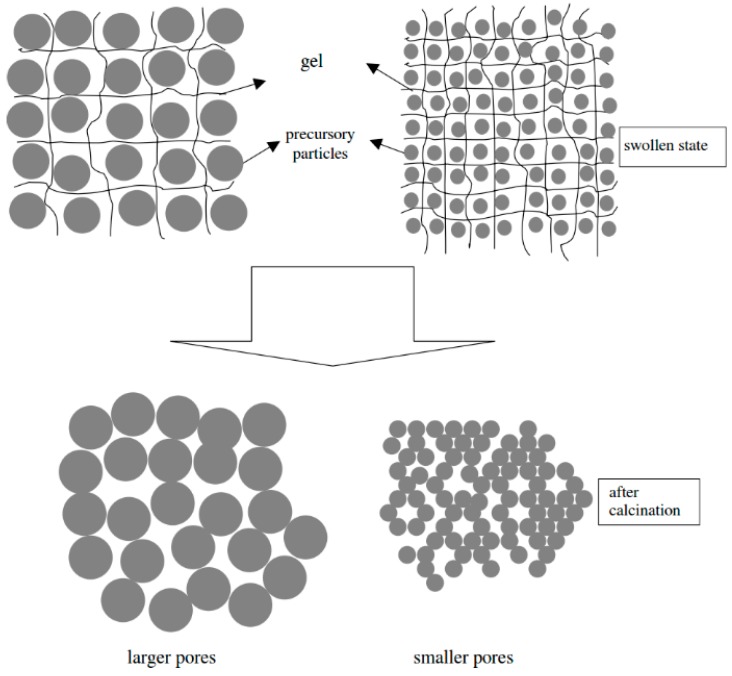
Potential impact of hydrogel mesh size on the porosity of the product. Reprinted with permission from [42], Copyright 2003 Elsevier.

Chemically cross-linked hydrogels may be regarded as the more obvious choice for utilization as porogenic matrices than physically cross-linked hydrogels. Chemical cross-linking may be more stable against potential de-mixing and phase separation during the growth of the inorganic phase. Moreover, the degree of cross-linking can be varied; by changing the density of cross-links it is possible to vary the ‘mesh size’ in the gel which, in return, will likely affect the porosity of the final product (Figure 2).

Kurumada et al. prepared hydrogels from hydroxyl ethyl methacrylate (HEMA) and ethylene glycol dimethacrylate (EGDMA) as a cross-linker and used them as porogenic matrices for SiO_2_, ZnO, and MgO [39,40,41,42]. Aqueous silica sols (from tetraethyl orthosilicate, TEOS) or methanolic solutions of Zn(NO_3_)_2_ or Mg(NO_3_)_2_, respectively, were used as the precursor systems and swelling agents. After conversion to the oxides and subsequent removal of the hydrogel matrices by thermal combustion, the porosity of the inorganic products varied depending on the mesh size, i.e., degree of crosslinking (HEMA/EGDMA ratio) in the hydrogel (Figure 2). Interestingly, contrary trends were observed for amorphous SiO_2_ [39] on the one hand, and ZnO [40] and MgO [41] on the other hand; high degrees of cross-linking resulted in smaller pores in case of silica (average pore sizes ranging from 3 to 16 nm; specific pore volumes from 0.3 to 0.65 cm^3^ g^−1^), but in larger pores for ZnO and MgO (pore volumes from 0.1 to 0.5 cm^3^ g^−1^). The authors suggested a model for the oxide formation in the hydrogel mesh to explain the different behavior [42]. The same group later used various polyacrylamide hydrogels as matrices for porous SiO_2_ [43,44,45]. They mixed two precursor solutions, one containing *N*-isopropylacrylamide (NIPAM) monomer and *N*,*N*’-methylenbisacrylamide (BIS) cross-linker, the other containing the inorganic precursor sol (from TEOS), to carry out the hydrogel formation (polymerization) and sol-gel transition (SiO_2_ formation) simultaneously [43,44]. The ratio of monomer to silica was varied and the smallest and most uniform pores (ca. 2 nm) were obtained for the lowest relative amount of monomer. The authors also varied the polarity of the hydrogel mesh by acrylamide monomers with different side chains (*N*,*N*’-dimethyl, *N*,*N*’-diethyl, *N*-isopropyl, *N*-isobutoxy) and found that higher polarity resulted in smaller pores [45].

Poly(2-hydroxyethyl methacrylate-*co*-acrylic acid) hydrogel films were used by Ford and Yang as matrices for porous amorphous SiO_2_ layers on silicon wafers [46]. Photo-induced cross-linking of the hydrogel took place after spreading the polymer at the wafer surface by spin-coating. This procedure allows for micropatterning of the film by masking during UV exposure. Poly(ethylenimine) brushes were grafted to the hydrogel backbone; their amino functions served as initiators for the polycondensation of silica under sol-gel conditions. After removal of the organic component SiO_2_ layers with thicknesses around 1 μm were obtained. The authors described the layers as consisting of SiO_2_ nanoparticles; the layer thickness varied, depending on the polyethylenimine (PEI) molecular weight. 

Bertozzi et al. have used a similar approach for the deposition and growth of hydroxyapatite in a poly(2-hydroxyethyl methacrylate) hydrogel matrix [47]. They used a urea-mediated precipitation technique that leads to hydrolysis of the 2-hydroxyethyl esters; the thus-created carboxylate functions promoted heterogeneous nucleation and 2-dimensional growth of calcium phosphate (CP). This study focused on biomimetic mineralization (for biocompatible bone-like composite materials), rather than attempting to create porosity by post-synthetic removal of the hydrogel matrix. Tong and Hu et al. employed a chemically cross-linked chitosan/glutaraldehyde hydrogel to prepare apatite; after removal of the hydrogel by oxidation with sodium hypochlorite, products with a certain degree of porosity were obtained [48].

**Figure 3:**
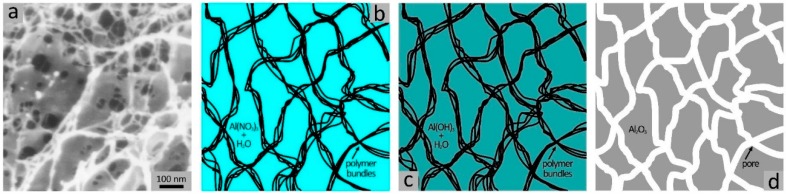
Schematic of the synthesis of mesoporous Al_2_O_3_ by using a dimethylacrylamide-based hydrogel as a porogenic matrix; (**a**) FESEM image of a hydrogel (shrunken state), (**b**) bundles of polymer strains in Al(NO_3_)_3_ solution, (**c**) after treatment with ammonia, and (**d**) after thermal decomposition of the polymer. Reprinted with permission from [49], Copyright 2014 Springer Nature.

We have recently reported the synthesis of mesoporous aluminum oxide (γ-Al_2_O_3_) by using photo-cross-linked dimethylacrylamide-based hydrogels as porogenic matrices [49,50]. The respective (non-cross-linked) polymers were swelled in a saturated aqueous solution of aluminum nitrate, followed by photo-induced cross-linking. Then the materials were dried and exposed to the vapor of an aqueous ammonia solution to convert aluminum nitrate to aluminum hydroxide. Finally, the materials were calcined to yield aluminum oxide and to simultaneously combust the hydrogel. The γ-Al_2_O_3_ products exhibited mesopore sizes in the range of 3 to 8 nm, large specific BET surface areas up to 360 m^2^ g^−1^, and specific pore volumes up to 0.50 cm^3^ g^−1^. Variation of the composition of the hydrogel matrix by adding various co-monomers in variable relative quantities turned out to have only little impact on the porosity of the products. The porogenic impact of the hydrogels was attributed to the presence of bundles of polymer strains that are incorporated in the inorganic phase during its precipitation and annealing (Figure 3). We also found that chemical cross-linking is not imperative for this synthesis concept; similar mesoporous Al_2_O_3_ and MgO materials were obtained by using the same kinds of polymers but without cross-linking [38]. (MgO exhibited lower porosity; its average pore size was 3.6 nm and the specific pore volume was 0.37 cm^3^ g^−1^.).

**Figure 4:**
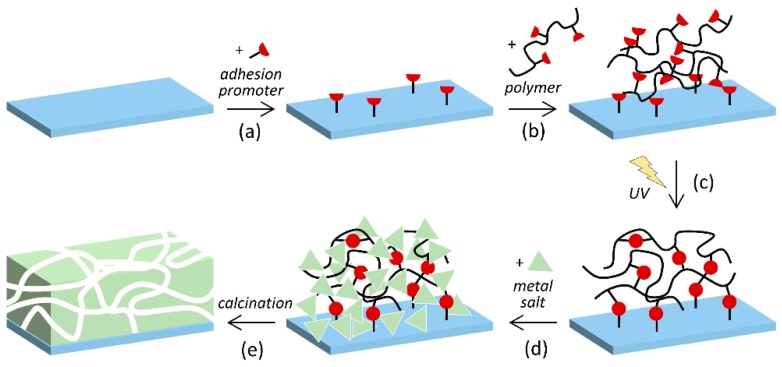
Schematic of the preparation of mesoporous metal oxide: (**a**) anchoring of an adhesion promoter on a substrate; (**b**) spreading of the polymer by spin coating; (**c**) hydrogel formation and immobilization on the substrate by photo-induced cross-linking; (**d**) swelling in metal salt solution; (**e**) formation of the porous metal oxide and combustion of the hydrogel by calcination [51].

**Figure 5:**
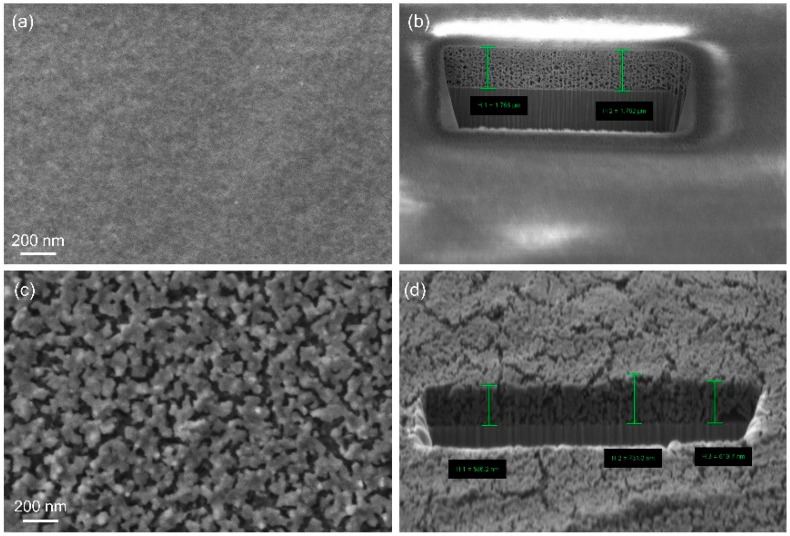
SEM images and focused ion beam (FIB) ablation analysis of mesoporous films of (**a**,**b**) Al_2_O_3_ (average thickness: 1.77 μm) and (**c**,**d**) MgO (average thickness: 0.59 μm) [51].

Utilization of hydrogel matrices offers the opportunity to prepare films and layers of porous materials by anchoring the hydrogel to the surface of a substrate (Figure 4). We have prepared poly(dimethylacrylamide) hydrogel films with sub-micrometer thicknesses (dry, non-swollen state) on silicon wafer substrates. The films were immobilized by means of a reactive chlorosilyl compound as an adhesion promoter; the promoter acts as a co-monomer in the polymerization and binds to the oxidized surface of the wafer by forming a siloxane bridge. Mesoporous Al_2_O_3_ and MgO were then prepared in the hydrogel film matrices. After combustion of the organic phase the inorganic products remained attached to the substrate, forming mesoporous layers with thicknesses of a few hundred nanometers (Figure 5) [51].

## 4. Further Examples of Hydrogel Matrices

Some authors have used hydrogels as matrices for the synthesis of individual nanoparticles. In these cases, the matrix does not exactly serve as a porogen, as the inorganic phase is formed only between adjacent meshes of the hydrogel, but does not incorporate the polymer strands. Subsequent removal of the polymer does therefore not yield pores in the inorganic product; rather, the role of the hydrogel network is to provide confined spaces (corresponding to the mesh size) for size-limited growth of the inorganic product. For example, Sahiner prepared various spherical metal (Fe, Co, Ni, Cu) and cadmium sulfide (CdS) particles in the sub-micron size range (‘quantum dots’) inside an anionic poly(2-acrylamido-2-methyl-1-propansulfonic acid) hydrogel [52]. The density of anionic functional groups in the hydrogel (binding the metal cations during the synthesis) had a clear influence on the particle size, whereas the mesh size turned out to have less impact. Hu et al. used a polyacrylamide hydrogel to synthesize cadmium selenide (CdSe) nanoparticles [53]. Likewise, Yan et al. prepared zeolite nanocrystals in a physically-cross-linked methylcellulose hydrogel [54].

## 5. Conclusions

In summary, physically or chemically cross-linked hydrogels are frequently used as matrices for the synthesis of inorganic materials. The hydrogels may initiate the nucleation of crystalline or amorphous phases and further direct their growth. The coordination abilities of the hydrogel atoms (especially N and O) influence the interactions with the metal ions and might be a possibility for tuning the structure of the resulting composite and the metal oxide, respectively. When the organic polymer strands are incorporated in the inorganic phase, their subsequent removal may leave behind mesopores, in which case the hydrogel acts as a porogen for the inorganic material. The removal of the organic phase (typically by thermal combustion) is a crucial point during the synthesis because the inorganic phase must be formed before the hydrogel starts to depredate. Otherwise, materials with a poor porosity might be obtained. Immobilization of the hydrogel matrix at a substrate surface facilitates the creation of uniform porous films and layers. By a selective chemical cross-linking it might be possible to create patterned structures, such as films, and utilize them in microfluidic devices, in catalysis or as a catalyst support. Since hydrogels can be modified in numerous respects, such as chemical composition, internal and external structure, the main challenges for future work will be to develop structure-property relationships between hydrogel structures, that can be controlled by synthesis protocols, and the final properties of the inorganic target structure.
